# Effect of Water Flooding on the Oviposition Capacity of Engorged Adult Females and Hatchability of Eggs of Dog Ticks: *Rhipicephalus sanguineus* and *Haemaphysalis leachi leachi*

**DOI:** 10.1155/2011/824162

**Published:** 2011-04-03

**Authors:** Johnson O. Adejinmi

**Affiliations:** Department of Veterinary, Microbiology and Parasitology, Faculty of Veterinary Medicine, University of Ibadan, Ibadan, Nigeria

## Abstract

Effects of water flooding on the oviposition capacity of engorged adult females and hatchability of eggs of *Rhipicephalus sanguineus* and *Haemaphysalis leachi leachi* under laboratory conditions were investigated. The durations of time of water flooding were 1, 2, 4, 6, 12, 24, 48, 72, 96, and 120 hours. Engorged females of *R. sanguineus* and *H. leachi leachi* did not oviposit after being flooded for more than 48 and 6 hours, respectively. The preoviposition periods of both species were longer than those of their controls. The number of eggs laid were significantly lower (*P* < .05) and higher (*P* < .05) than their controls, respectively, for *R. sanguineus* and *H. leachi leachi* flooded for 1–4 hours. The hatchability of eggs of both species decreased as flooding time increased. The percentage of hatchability was negatively correlated with flooding time and was highly significant (*r* = −0.97; *P* < .10). It is concluded that *R. sanguineus* tolerated simulated water flooding more than *H. leachi leachi*.

## 1. Introduction

The importance of ticks as parasites of domesticated animals lie in their ability to successfully multiply and establish in their environment and their ability to transmit diseases to their hosts and to maintain their physiological requirements [1]. A number of extrinsic factors affect the successful reproductive performance and survival of ixodid ticks after detachment from their hosts [2, 3]. Such extrinsic factors include rainfall, humidity, temperature, floods, winds, and physical damage [3, 4].

 The response of the ticks to these factors, to a large extent, determines their preponderance and abundance in the environment.

Earlier works [3, 5–7] have shown that the season of the year and the field environment in which the ticks oviposit are important extrinsic factors as ticks were observed to produce greater number of eggs during the peak and end of rains and when placed in shade. It has been noted by some workers [5, 8, 9] that while success in oviposition was aided by moisture provided by light-to-moderate rainfall, excessive moisture in terms of heavy rainfall adversely affected tick oviposition and distribution. As observed by Hinton [10], it is not fully understood how terrestrial arthropods survive the temporary inundations that follow heavy rains. 

Murray and Vestjens [11] and Daniel and Gerry [12] attributed the scarcity of or complete absence of ixodid ticks from certain habitats to periods of excessive wetness. Some workers have investigated effects of water flooding with varying results. Sutherst [13] in his study of effect of flooding on the ixodid tick *Boophilus microplus* in Australia, found that eggs and larvae of *B. microplus* were more resistant to submersion than engorged female and that their survival was increased at low temperatures and in water with high oxygen content. He also noted that the egg production of *B. microplus* was reduced significantly only after submersion for 24 hours. The author then suggested that the effect of heavy rain is the silting up of egg masses.

Amoo [14] in his work on cattle tick *Boophilus* species found that the vitality of eggs of *Boophilus decoloratus* and *B. geigyi* flooded with water for up to 48 hours was not affected and that their engorged females failed to lay eggs after flooding with water for 24 and 72 hours, respectively.

Dipeolu et al. [6] investigated extrinsic factors influencing oviposition and egg-hatch of *Amblyomma variegatum* under natural conditions and observed two types of oviposition pattern. These authors reported that adult *A. variegatum* engorged to more than 2.49 grams were affected by immersion in water for longer than 7 days, and that such ticks died without ovipositing and the water in which they were submerged became dark red. The authors also reported that eggs immersed in water ranging from 1–7 days hatched in about the same number of days as control eggs, and their viability in terms of percent-hatch was not affected. To date there is paucity of information on the effect of water flooding on ticks infesting dogs in Nigeria: *R. sanguineus *and *H. leachi leachi.*

This paper reports the oviposition capacity of engorged adult females and performance of eggs of *R. sanguineus *and *H. leachi leachi *when subjected to simulated field flooding in the laboratory. The knowledge from this study will help in understanding the population dynamics of these ticks in the rain forest areas where there are heavy rains.

## 2. Materials and Methods

The ticks used for this investigation were adult females in various stages of engorgement collected individually by careful detachment with pairs of forceps from household dogs brought to veterinary clinics in Ibadan. The ticks were collected into glass bottles and conveyed in kilner jars to parasitology laboratory in the Department of Veterinary Microbiology and Parasitology, University of Ibadan, identified and individual weights were determined and recorded using a sensitive Sartorius balance (Type 2472).

Five engorged females of the same weight each of *R. sanguineus *and *H. leachi leachi *were put in Bijou bottles, filled with tap water (with unknown contaminants) for varying durations of time: 1, 2, 4, 6, 12, 24, 48, 72, 96, and 120 hours. After each duration of time the water was decanted, the ticks were dried with Whatman filter paper, put in dry clean Bijou bottles, plugged with cotton wool, and incubated at 25°C and 85% relative humidity (RH) as described by Amoo [14] and Dipeolu et al. [6]. Another set of five ticks each of *R. sanguineus *and *H. leachi leachi *which were not flooded with water served as the controls. The ticks were observed daily for oviposition until the end of last oviposition. The preoviposition and oviposition periods and the number of eggs laid were recorded. The eggs laid by five ticks from each time regimen were pooled together.

Effect of water flooding on eggs was carried out by filling bijou bottles containing 0.05 gram freshly laid eggs each of *R. sanguineus *and *H. leachi leachi *with tap water for varying durations of time: 1, 2, 4, 6, 12, 24, 48, 72, 96, and 120 hours. After duration of each time, the eggs were recovered by decanting the water and putting them on Whatman filter paper as described earlier [6, 14]. Thereafter, the eggs were returned in another clean and dry Bijou bottles into the incubator maintained at 25°C and 85% RH.

Another set of 0.05 gram freshly laid eggs each of *R. sanguineus *and *H. leachi leachi *which were not flooded with water but were also incubated at 25°C and 85% RH and served as controls. The eggs were observed daily for hatching till the end of hatching of eggs and two weeks after the last larva (e) have hatched out of the eggs. From the day hatching started until it ended the hatched larvae were separated at 8.00 hour every morning from the unhatched eggs. This was possible because before hatching the eggs usually clumped together. Once hatching started the larvae move away from the clump. The separation of larvae from unhatched eggs was therefore achieved by scooping the lump(s) of unhatched eggs into another bijou bottle which was then covered tightly, while 10% formalin was added to the first bijou bottle which then contained only hatched larvae [15].

The number of larvae was ascertained under a dissecting microscope. This was repeated every day until no larva was seen to hatch. The unhatched eggs which existed in small clumps were observed for another two weeks before they were declared unhatched and dead. They were then taken out of bijou bottle and counted through the addition of xylene under the dissecting microscope [16]. The eclosion period, duration of hatching, and percentage hatchability were recorded.

All data were subjected to analyses of variance (ANOVA), Chi-square test, Student's *t*-test and correlation coefficient using the computer package SPSS version 1.12002.

## 3. Results

Table 1 shows the effect of water flooding on the oviposition capabilities of engorged adult females of *R. sanguineus *and *H. leachi leachi. *Engorged females of *R. sanguineus *did not oviposit after being flooded for more than 48 hours. Also engorged adult females of *H. leachi leachi* did not oviposit when flooded for more than six hours. The preoviposition periods of engorged females of both species were longer than those of their controls which were not given water treatment. 

The oviposition periods of *R. sanguineus* and *H. leachi leachi* flooded for 1–4 hours were shorter and longer than their control, respectively.

Also the number of eggs laid were significantly lower (*P* < .05) and higher (*P* < .05) than the control, respectively, for* R. sanguineus* and *H. leachi leachi* flooded for the same period of time. 

**Figure 1:**
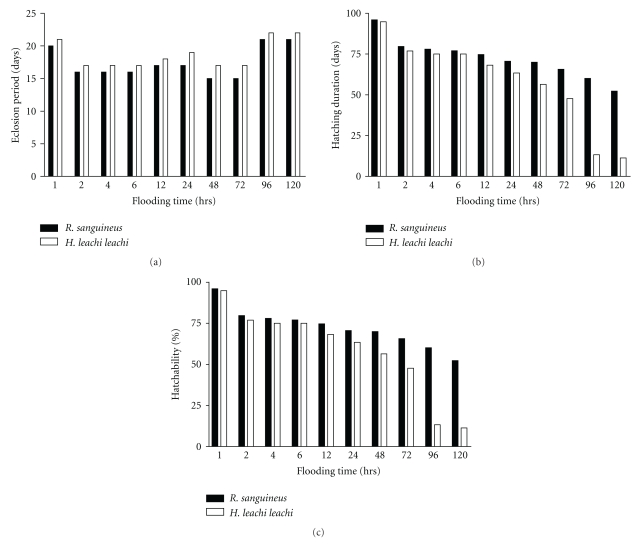
(a) The eclosion period of eggs of* R. sanguineus* and *H. leachi leachi* at different flooding time. (b) The duration of hatching of eggs of *R. sanguineus* and *H. leachi leachi* at different flooding time. (c) The hatchability of eggs of *R. sanguineus* and *H. leachi leachi *at different flooding time.

Eggs of both *R. sanguineus *and *H. leachi leachi *kept flooded between 2 and 120 hours hatched with significant difference (*P* < .05) compared with their controls (Figures 1(a)–1(c)). In both species hatchability decreased as flooding time increased. The percentage hatchability was negatively correlated with flooding time and was highly significant (*r* = −0.97, *P* < .01). The results also show that the durations of hatching, for eggs of *R. sanguineus *and *H. leachi leachi* flooded for different periods between 1 and 120 hours were longer than those of their respective controls. The eclosion periods of eggs of both species decreased between 1 and 48 hours and increased between 72 and 120 hours.

## 4. Discussion

The results of this study on the oviposition capacity of adult females of *R. sanguineus *and *H. leachi leachi* agree with the findings of Sutherst [13] who reported that egg production of *B. microplus* was reduced significantly after submersion for 24 hours, indicating that their metabolism was not affected prior to this time. The results are also in agreement with the observations of Amoo [14] who reported that the engorged females of *B. decoloratus* and *B. geigyi* failed to lay eggs after flooding with water for longer than 24 and 72 hours, respectively. 

 The results however contrast sharply with the findings of Dipeolu et al. [6] in their study of *Amblyomma variegatum*. These authors reported that excessive water is not inimical to the viability of engorged adults and eggs of *A. variegatum* if they are not subjected to flooding longer than 7 days.

From the results of this investigation engorged adult females of *R. sanguineus *tolerated simulated water flooding more than the adult females of *H. leachi leachi.* These may explain the preponderance of these key species of ticks infesting dogs in this area, a forest zone with heavy rainfall, where *R. sanguineus* is more abundant than *H. leachi leachi* [17, 18].

According to Sutherst [13], flood rains would cause only a temporary reduction in tick numbers and the long-term effect of the rains is to create environmental conditions that are highly favourable for tick reproduction. Hinton [19] described the size and form of the aeropyles or spiracles of *Boophilus.* This author stated that the main function of the aeropyles is to block entry of water under field conditions. Since the duration of action of these aeropyles differ from one tick species to the other, it is reasonable to suggest that the action of the aeropyles is only effective within the first 6 hours, for *H. leachi leachi *and longer up to 48 hours for *R. sanguineus. *Thus, the structure of the spiracles of ixodid ticks, especially those which spend much of their life cycle close to the ground, may therefore be an adaptation which enables them to survive temporary inundations during the rainy season. Also, the prolonged preoviposition period of adult female ticks of both species, flooded for more than 2 hours might be a resting or recovery period following periods of anoxia, as has been observed for *B. microplus *[13] and for *B. geigyi *and *B. decoloratus *[14]. 

The results on the hatchability of eggs of *R. sanguineus* and *H. leachi leachi* however contrast sharply with that of Amoo [14] who observed that the eggs of *B. decoloratus* and *B. geigyi* immersed in water for longer than 48 hours failed to hatch.

The results also disagree with the report of Dipeolu et al. [6] that eggs of *A. variegatum* immersed in water ranging from 1–7 days hatched in about the same number of days as control eggs and their viability in terms of percent-hatch was not affected.

 The results of investigation into the hatchability of eggs of *R. sanguineus *and *H. leachi leachi* after water flooding show that flooding up to 5 days did not affect their survival and suggests that flooding would have to be very prolonged for more than 5 days to have a deleterious effect on the eggs. The results on the hatchability of eggs confirm the observation of Sutherst [13] who reported that the greatest danger to eggs of ixodid ticks on the field would be the silting up of egg masses during heavy rains. This is because the egg when immersed in water could obtain oxygen from the water and so unlike adults were not subjected to anoxia while in aerated water. The significantly (*P* < .05) shorter eclosion periods and decrease hatchability of eggs of *R. sanguineus* and *H. leachi leachi * flooded between 1 and 48 hours compared with their controls observed in this study could be explained in terms of osmosis. Since the water with which the eggs were flooded was hypotonic to the contents of the eggs which include proteins, carbohydrate, and lipids [20], water will be drawn into the eggs making them to swell and burst. In addition to, this protein and enzymes within the egg may become coagulated and hence rendered inactive with consequent negative effect on egg-hatch. The longer eclosion periods and decrease hatchability of eggs of both tick species flooded between 72 and 120 hours compared with their controls observed in this study are not clear and are being investigated further in our laboratory.

## 5. Conclusion

It is therefore concluded that both the adult females and eggs of *R. sanguineus* tolerated simulated water flooding more than those of *H. leachi leachi,* and this probably explains why *R. sanguineus* is more preponderant and abundant than *H. leachi leachi* in Ibadan. However, the two species would survive the prolonged heavy rainfall usually experienced in the forest zones of Nigeria, since flood rains would cause only a temporary reduction in tick numbers on the short term and create conditions that are favourable for tick reproduction on the long term.

## Figures and Tables

**Table 1:** Effect of water flooding on the oviposition capacity of engorged adult females of *Rhipicephalus sanguineus* and *Haemaphysalis leachi leachi. *

Flooding time (Hrs.)	*R. sanguineus*			*H. leachi leachi*		
	Mean Preoviposition period (days)	Mean duration of oviposition (days)	Mean no. of eggs laid	Mean preoviposition period (days)	Mean duration of oviposition (days)	Mean no. of eggs laid
Control 0	5.0 ± 0	9.33 ± 1.15	1402.67 ± 128.52	3.0 ± 0	8.33 ± 1.15	857.33 ± 203.89
1	6.33 ± 0.58	5.67 ± 1.53	279.67 ± 4.51	8.0 ± 0.58	12.67 ± 1.15	968.33 ± 18.01
2	7.0 ± 0	8.67 ± 0.58	975.0 ± 5.0	8.33 ± 0.58	12.67 ± 0.58	1557.0 ± 32.91
4	7.33 ± 0.58	9.00 ± 0	1032.67 ± 62.66	9.0 ± 0	11.33 ± 0.58	1069.33 ±5.03
6	7.0 ± 0	10.33 ± 0.58	1464.33 ± 6.03	10.33 ± 0.58	13.0 ± 1.0	1074.33 ± 67.04
12	8.33 ± 0.58	10.0 ± 0	1663.67 ± 4.16	—	—	—
24	8.67 ± 0.58	10.0 ± 0	1116.0 ± 4.0	—	—	—
48	11.33 ± 0.58	10.67 ± 0.58	1506.33 ± 0.51	—	—	—
72	—	—	—	—	—	—
96	—	—	—	—	—	—
120	—	—	—	—	—	—
